# The use of methylphenidate in vascular dementia: A case report

**DOI:** 10.1192/j.eurpsy.2021.1136

**Published:** 2021-08-13

**Authors:** P. Regueira, J. Cerejeira

**Affiliations:** Department Of Psychiatry, Centro Hospitalar e Universitário de Coimbra, Coimbra, Portugal

**Keywords:** methylphenidate, Therapeutic approach, Vascular dementia

## Abstract

**Introduction:**

Patients diagnosed with vascular dementia often present with apathy, executive dysfunction or/and memory impairment. Some of these psychiatric domains are not responsive to antidepressants or acetylcholinesterase inhibitors. Since methylphenidate enhances frontal lobe function, it may be a valid therapeutic option.

**Objectives:**

To report a case where methylphenidate was used as a therapeutic approach in vascular dementia.

**Methods:**

We present a case of a patient diagnosed with vascular dementia with substantial clinical improvement after treatment with methylphenidate.

**Results:**

A 67 year-old male was observed in a psychiatric consultation reporting memory loss, inability to retain information and inattention. According to her spouse, the patient has been mostly isolated at home and recently he has become unable to accomplish some daily living activities. There was no history of previous psychiatric disorder. Cognitive assessment was performed using MoCA test: 19/30 points (predominantly in executive, attention and delayed recall domains). After this evaluation, it was introduced bupropion 150mg od and donepezil 5mg od with insignificant clinical improvement. The patient underwent a routine workup which was unremarkable and a brain computed tomography scan that revealed ischemic leukoencephalopathy. Three months later no clinical benefit was reported. Attention and functional improvement were observed after introduction of methylphenidate with progressive dose adjusting till 30 mg/day.

**Conclusions:**

Besides not being a consensual therapeutic approach, considering that there is a lack of efficient pharmacological strategies in vascular dementia, methylphenidate may play a significant role in this field contributing to clinical improving and ultimately to an enhanced quality of life.

